# Characterizing medical patients with delirium: A cohort study comparing ICD-10 codes and a validated chart review method

**DOI:** 10.1371/journal.pone.0302888

**Published:** 2024-05-13

**Authors:** Kathleen A. Sheehan, Saeha Shin, Elise Hall, Denise Y. F. Mak, Lauren Lapointe-Shaw, Terence Tang, Seema Marwaha, Dov Gandell, Shail Rawal, Sharon Inouye, Amol A. Verma, Fahad Razak

**Affiliations:** 1 Department of Psychiatry, University of Toronto, Toronto, ON, Canada; 2 Centre for Mental Health, University Health Network, Toronto, ON, Canada; 3 St. Michael’s Hospital, Unity Health Network, Toronto, ON, Canada; 4 Department of Psychiatry, Unity Health Network, Toronto, ON, Canada; 5 Department of Medicine, University of Toronto, Toronto ON, Canada; 6 Department of Medicine, University Health Network, Toronto, ON, Canada; 7 Institute for Better Health, Trillium Health Partners, Mississauga, ON, Canada; 8 Department of Medicine, Unity Health Network, Toronto, ON, Canada; 9 Department of Medicine, Sunnybrook Heatlh Sciences Centre, Toronto, ON, Canada; 10 Aging Brain Center, Hebrew Senior Life, Boston, MA, United States of America; 11 Beth Israel Deaconess Medical Center, Harvard Medical School, Boston, MA, United States of America; Center for Primary Care and Public Health: Unisante, SWITZERLAND

## Abstract

**Background:**

Delirium is a major cause of preventable mortality and morbidity in hospitalized adults, but accurately determining rates of delirium remains a challenge.

**Objective:**

To characterize and compare medical inpatients identified as having delirium using two common methods, administrative data and retrospective chart review.

**Methods:**

We conducted a retrospective study of 3881 randomly selected internal medicine hospital admissions from six acute care hospitals in Toronto and Mississauga, Ontario, Canada. Delirium status was determined using ICD-10-CA codes from hospital administrative data and through a previously validated chart review method. Baseline sociodemographic and clinical characteristics, processes of care and outcomes were compared across those without delirium in hospital and those with delirium as determined by administrative data and chart review.

**Results:**

Delirium was identified in 6.3% of admissions by ICD-10-CA codes compared to 25.7% by chart review. Using chart review as the reference standard, ICD-10-CA codes for delirium had sensitivity 24.1% (95%CI: 21.5–26.8%), specificity 99.8% (95%CI: 99.5–99.9%), positive predictive value 97.6% (95%CI: 94.6–98.9%), and negative predictive value 79.2% (95%CI: 78.6–79.7%). Age over 80, male gender, and Charlson comorbidity index greater than 2 were associated with misclassification of delirium. Inpatient mortality and median costs of care were greater in patients determined to have delirium by ICD-10-CA codes (5.8% greater mortality, 95% CI: 2.0–9.5 and $6824 greater cost, 95%CI: 4713–9264) and by chart review (11.9% greater mortality, 95%CI: 9.5–14.2% and $4967 greater cost, 95%CI: 4415–5701), compared to patients without delirium.

**Conclusions:**

Administrative data are specific but highly insensitive, missing most cases of delirium in hospital. Mortality and costs of care were greater for both the delirium cases that were detected and missed by administrative data. Better methods of routinely measuring delirium in hospital are needed.

## Introduction

Delirium is a common and treatable neurocognitive disorder, characterized by acute onset of change in mental status, psychomotor disturbance and hallucinations [1]. It affects up to 50% of hospitalized adults over the age of 65, is potentially preventable, and is associated with numerous negative outcomes in hospital including increased mortality, longer stays, and higher health care costs [2,3]. Post-discharge, it is associated with nursing home placement, declines in cognitive function, and incident dementia [4–6]. Given its great clinical and economic burden, improving prevention and management of delirium is increasingly of interest and delirium is now widely recognized as an indicator of healthcare quality [7–11]. However, diagnosing and measuring rates of in-hospital delirium remains a challenge. Tracking the impact of quality improvement initiatives and the conduct of system-wide evaluation are therefore difficult.

Delirium rates published in the literature are obtained through a variety of methods. Administrative data capture the rate of delirium diagnosis, chart review estimates the rate of delirium based on chart documentation, and clinical assessment determines the incidence or prevalence of delirium from signs, symptoms, and collateral information. Given its lower cost and ready accessibility, administrative data has been recommended for evaluating delirium rates according to quality standards in Australia and Canada [9,10]. This method is also used in population-based and health services research [12,13]. However, it is recognized that administrative data substantially underestimate the frequency of delirium compared to case ascertainment by either chart review or clinical assessment [12,14–22]. While some studies have compared administrative coding to clinical assessment, there are limited data comparing administrative codes to a validated chart review method [17–19,23]. Moreover, these studies have focused on post-surgical populations with limited data from general medical inpatients. From a system perspective, understanding who is, or is not, captured by each method may be of more use than understanding the accuracy of each detection method. With this knowledge, those working in healthcare institutions can decide which method of delirium identification is most appropriate for their aims when implementing quality assurance and improvement interventions to prevent delirium. In this study, we aimed to characterize and compare the populations of medical inpatients with delirium, as identified by administrative data and a chart review method.

## Method

### Design, setting and study population

We conducted a retrospective chart review study, using the CHART-DEL method ^23^. We randomly selected 3881 hospital admissions from four healthcare institutions (six hospitals) in Toronto, Ontario. Participating institutions were St. Michael’s Hospital (SMH), University Health Network (UHN: Toronto General Hospital and Toronto Western Hospital), Sunnybrook Health Sciences Centre (SBK), and Trillium Health Partners (THP: Credit Valley Hospital and Mississauga Hospital). Each hospital is part of the GEMINI research network which collects administrative and clinical data on all adult (>18 year of age) patients who are admitted to or discharged from general medicine services [24,25]. These hospitals range in size from 433 to 1325 acute inpatient beds, with general internal medicine (GIM) patients accounting for 24% of hospital bed-days and 39% of admissions through the emergency department during the study period of April 1 2010 –March 20 2015 [24]. Ontario has a publicly funded health care system that insures residents for medically necessary services provided in hospital settings.

### Data sources

GEMINI collects and links administrative and clinical data for patients admitted to or discharged from general medicine services [24,26]. The administrative data include information reported by participating hospitals to the Canadian Institute for Health Information (CIHI) Discharge Abstract Database (DAD): demographic characteristics, diagnoses, interventions, discharge destinations, and resource use. For each hospitalization up to 25 diagnoses are recorded at the time of discharge in the DAD.

The CHART-DEL method, which has a sensitivity of 74% and specificity of 83% compared to clinical assessment, was used to identify delirium during hospitalization [23]. Information about and documentation of acute changes in mental status were transcribed verbatim, as well as improvements in mental state.

### Chart-based delirium identification

Trained abstractors, who all had clinical experience, used the CHART-DEL method to code for the presence of delirium during admission to hospital [23]. As outlined in the CHART-DEL training guide, all abstractors participated in the recommended training procedure [27]. This included didactic sessions on delirium and the chart review method. Initial chart abstractions were performed jointly and each abstractor then reviewed ten charts independently, which were compared to an expert rater (EH, KAS, DG, TT) to ensure accuracy and reliability. Abstractors reviewed the entire chart, including daily progress notes by physicians and nursing staff, assessments by allied health professionals such as occupational and physical therapy, specialist physician consultations, and admission and discharge notes. They were blinded to administrative data coding of delirium. If abstractors were unable to determine whether delirium was present, the case was labelled as uncertain and then reviewed by expert raters (experienced physicians in internal medicine and consultation-liaison psychiatry with specialized interest in delirium) at each institution to make a final determination. Chart data were accessed between August 6 2016 and December 20 2019. Authors did not have access to information that could identify individual participants during or after data collection.

### Administrative data delirium identification

Administrative data were generated by hospital-based chart coders who review the patient chart post-discharge and code diagnoses using the enhanced Canadian version of the 10^th^ revision of the International Statistical Classification of Diseases and Related Health Problems (ICD-10-CA). This is a standard process completed for all patients discharged from hospital in Canada. Hospital coders did not have access to study data. Given challenges with delirium nomenclature, we used both narrow (codes that include delirium related and unrelated to substances) and broad definitions (codes that include encephalopathy) of delirium in administrative data [18,28] (see S1 Appendix). In contrast to the United States, there is no billing incentive for encephalopathy compared to delirium and the broad definition only identified three additional cases, which were not analysed further.

### Variables

Administrative health data were linked with clinical data extracted from hospital information systems [24]. Demographic variables included age and gender. Clinical variables included the laboratory-based acute physiology score (LAPS), Charlson comorbidity index (CCI), and a pre-admission diagnosis of dementia [29–31]. Processes of care variables included length of stay, admission to an intensive care unit (ICU), and ICU length of stay (LOS). Outcome variables included total cost of hospitalization (direct and indirect costs from the emergency department and inpatient portions of the hospital visit), mortality, 30-day readmission to general internal medicine (GIM) at any participating hospital, new diagnosis of dementia at discharge, and a new discharge destination of a long term care (LTC) facility [32].

In addition to variables extracted from these electronic systems, processes of care variables were also coded during the process of manual chart review. These included specialist physician consultation (geriatric medicine, psychiatry, and geriatric psychiatry), allied health (physiotherapy and occupational therapy) assessment, and completion of neurocognitive screening.

### Statistical analysis

The sample size was selected based on the feasibility of chart review, with an estimated 30–60 minutes required per admission. Chart-based identification of delirium was compared with administrative data identification by calculating sensitivity, specificity, positive predictive value, and negative predictive value. The chart-based method was used as the reference standard. Descriptive statistics were used to compare demographic, clinical, service use, and outcome variables between those identified by chart review and those identified through administrative data. Given the sample size, standardized differences (SD) were used to assess statistical significance, with differences of greater than 0.1 considered significant [33]. Baseline factors associated with incorrect identification of delirium by administrative data (false positives and false negatives) were examined using unadjusted and adjusted logistic regression models, including patient demographic and clinical variables. A random subsample of 5% of charts were coded by two abstractors to calculate inter-rater reliability using a kappa score.

### Ethics approval

Ethics approval was obtained from the research ethics board of all participating hospitals (St. Michael’s Hospital, University Health Network, Sunnybrook Health Sciences Centre, Trillium Health Partners), with St. Michael’s Hospital as the Board of Record and a waiver of patient consent for this retrospective study using routinely collected health data.

## Results

**Fig 1:**
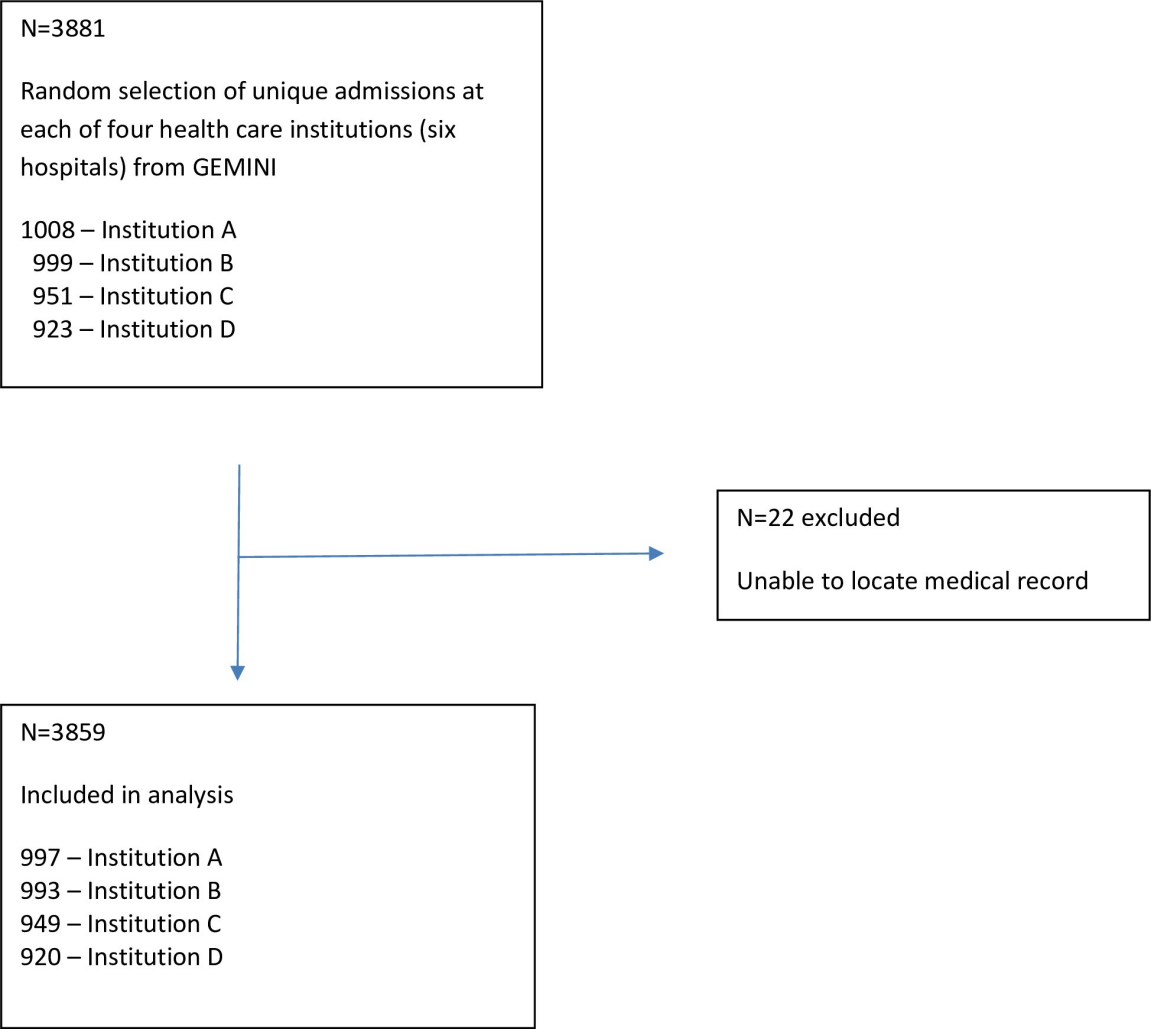
Study sample.

Of the 3881 randomly selected patient records, the final sample included 3859 patient admissions across six hospitals. Twenty-two were excluded because we were unable to locate patient records (Fig 1). Interrater reliability on the double-coded charts (5% sample, N = 193) was high, with kappa = 0.9 for presence or absence of delirium.

As shown in Table 1, the sample was 49.6% male and the median age was 73 years (IQR: 57–84). The mean LAPS was 20.48 (SD: 17.56). Similar proportions of the sample had either low (41.5%, score of 0) or high (42.3%, score >2) levels of comorbidity as measured by the Charlson comorbidity index. Approximately 2% (N = 67) had dementia noted as a pre-admission comorbidity [31]. Delirium was identified in 992 (25.7%) patients by chart review, compared to 245 (6.3%) using ICD-10 codes. In only six cases (0.16%) was delirium identified by ICD-10 codes, but not by chart review (S2 Appendix shows sample characteristics by chart review delirium identification). The use of the chart review method detected 753 more patients than those identified by ICD-10 codes alone.

**Table 1:** Identified delirium and association with baseline characteristics.

	Overall Sample	No delirium	Delirium by ICD-10-CA	Delirium by chart review (CR)	Delirium by chart review only (CR-only)	95% CI No delirium vs. ICD-10-CA	95% CI No delirium vs CR	95% CI ICD-10-CA vs CR
N	3859	2861	245	992	753			
Age Median (IQR)	73.0 (57.0, 84.0)	69.0 (76.0, 89.0)	83.0 (76.0, 89.0)	80.00 (69.00, 87.00)	79.0 (67.0, 87.0)	14 (12, 16)	11 (10, 13)	3 (1, 5)
Gender Male (%)	1913 (49.6)	1403 (49.0)	104 (42.4)	507 (51.1)	406 (53.9)	6.6 (0.1, 13.1)	2.1 (-1.5, 5.7)	8.7 (1.7, 15.6)
LAP Score Mean (SD)	20.48 (17.56)	18.78 (16.53)	22.15 (15.46)	25.41 (19.42)	26.39 (20.45)	3.4 (1.3, 5.4)	6.6 (5.3, 8.0)	3.3 (5.6, 9.8)
CCI (%) 0 1 2+	1602 (41.5) 624 (16.2) 1633 (42.3)	1293 (45.2) 485 (17.0) 1083 (37.9)	71 (29.0) 40 (16.3) 134 (54.7)	309 (31.1) 139 (14.0) 544 (54.8)	238 (31.6) 99 (13.1) 416 (55.2)	16.2 (10.2, 22.2) 0.7 (-4.2, 5.5) 16.8 (10.3, 23.4)	14.1 (10.5, 17.4) 3.0 (0.3, 5.5) 16.9 (13.3, 20.4)	2.1 (-4.2, 8.6) 2.3 (-2.8, 7.5) 0.1 (-6.9, 7.1)
Pre-Admission Dementia (%)	67 (1.7)	34 (1.2)	14 (5.7)	33 (3.3)	19 (2.5)	4.5 (1.6, 7.5)	2.1 (1.0, 3.3)	2.4 (-0.7, 5.5)

CR = chart review using CHART-DEL; ICD-10-CA = ICD-10-CA administrative data coding; IQR = inter-quartile range; SD = standard deviation; CI = confidence interval; LAP = laboratory-based acute physiology; CCI = Charlson comorbidity index.

Tables 1–3 compare baseline, process, and outcome data across four groups: those identified as not having delirium by both methods (no delirium, n = 2861), those identified as having delirium by ICD-10 codes (n = 245), those identified as having delirium by chart review (n = 992), and those identified by chart review but not ICD-10 codes (n = 753). Delirium was associated with older age, greater LAPS, greater Charlson comorbidity index, and having a pre-admission diagnosis of dementia (Table 1). Patients with delirium identified by either method were more likely than patients without delirium to have assessments completed by occupational therapy, physiotherapy, geriatric medicine or psychiatry, and have cognitive testing completed (Table 2). Those with delirium identified by ICD-10 codes were more likely to have these assessments performed than those identified by chart review alone. Hospital length of stay and costs of care were greater for patients identified as having delirium by ICD-10 codes (median 11.45 days, IQR 5.83–22.17; median $11,023.60, IQR 5,125.00–22,223.10) or chart review (median 8.8 days, IQR 4.5–17.4; median $9,180.00, IQR 4,552.60–19,411.00) compared to patients without delirium (median 4.08 days, IQR 2.04–7.62; median $4,199.30, IQR 2,290.45–7,885.79). Similarly, inpatient mortality and ICU admission were greater in patients with delirium identified by ICD-10 codes (mortality 9.4%, ICU 10.2%) or chart review (mortality 15.5%, ICU 15.5%) compared to patients without delirium (mortality 3.6%, ICU 5.6%). A new diagnosis of dementia and new placement in long-term care were also greater in patients with delirium identified by either method compared to patients without delirium (Table 3).

**Table 2:** Identified delirium and association with processes of care.

	Overall Sample	No delirium	Delirium by ICD-10-CA	Delirium by chart review (CR)	Delirium by chart review only (CR-only)	95% CI No delirium vs. ICD-10-CA	95% CI No delirium vs CR	95% CI ICD-10-CA vs CR
N	3859	2861	245	992	753			
OT Assessment	1266 (32.8)	746 (26.1)	167 (68.2)	515 (51.9)	353 (46.9)	42.1 (36.0, 48.2)	25.8 (22.2, 29.2)	16.3 (9.6, 22.9)
PT Assessment	1536 (39.8)	941 (32.9)	179 (73.1)	591 (59.6)	416 (55.2)	40.2 (34.3, 46.0)	26.6 (23.1, 30.1)	13.5 (7.1, 19.9)
Geriatric Medicine Consult	205 (5.3)	75 (2.6)	74 (30.2)	128 (12.9)	56 (7.4)	27.6 (21.8, 33.4)	10.2 (8.1, 12.4)	17.3 (11.2, 23.5)
Geriatric Psychiatry Consult	77 (2.0)	39 (1.4)	19 (7.8)	37 (3.7)	19 (2.5)	6.4 (3.0, 9.8)	2.3 (1.1, 3.6)	4.1 (0.5, 7.6)
Psychiatry Consult	189 (4.9)	109 (3.8)	22 (9.0)	78 (7.9)	58 (7.7)	5.2 (1.5, 8.8)	4.0 (2.2, 5.8)	1.1 (-2.9, 5.1)
Cognitive Testing Performed	361 (9.4)	165 (5.8)	71 (29.0)	195 (19.7)	125 (16.6)	23.2 (17.4, 29.0)	13.9 (11.3, 16.5)	9.3 (3.1, 15.6)
ICU Admission (%)	315 (8.2)	160 (5.6)	25 (10.2)	154 (15.5)	130 (17.3)	4.6 (0.7, 8.5)	9.9 (7.5, 12.3)	5.3 (0.9, 9.8)
ICU LOS (days) Median (IQR)	3.96 (1.66, 8.02)	2.69 (1.49, 6.22)	6.00 (4.25, 10.72)	5.3 (2.0, 10.8)	4.45 (1.79, 10.75)	3.3 (-1.7, 7.0)	2.6 (1.8, 3.6)	0.7 (-3.7, 5.1)

Chart review = CHART-DEL; ICD-10-CA = ICD administrative data coding; IQR = inter-quartile range; SD = standard deviation; CI = confidence interval; OT = occupational therapy; PT = physiotherapy; ICU = intensive care unit; LOS = length of stay in days; CAD = Canadian dollar currency; AMA = against medical advice; GIM = general internal medicine; LTC = long term care facility.

**Table 3:** Identified delirium and association with outcomes.

	Overall Sample	No delirium	Delirium by ICD-10-CA	Delirium by chart review (CR)	Delirium by chart review only (CR-only)	95% CI No delirium vs. ICD-10-CA	95% CI No delirium vs CR	95% CI ICD-10-CA vs CR
N	3859	2861	245	992	753			
LOS (days) Median (IQR)	4.85 (2.50, 9.61)	4.08 (2.04, 7.62)	11.45 (5.83, 22.17)	8.8 (4.5, 17.4)	8.34 (4.42, 16.50)	7.4 (5.3, 9.0)	4.7 (4.0, 5.5)	2.7 (0.4, 4.3)
Total cost (CAD) Median (IQR)	$5 056.84 (2652.24, 9998.19)	$4 199.30 (2290.45, 7885.79)	$11 023.60 (5125.00, 22 223.10)	9 180.0 (4552.6, 19411.0)	$9 014.00 (4372.00, 18350.00)	6824 (4713, 9264)	4967 (4415, 5701)	1844 (-602, 4019)
Mortality (%)	259 (6.7)	104 (3.6%)	23 (9.4%)	154 (15.5%)	132 (17.5%)	5.8 (2.0, 9.5)	11.9 (9.5, 14.2)	6.1 (1.8, 10.5)
30 Day GIM Re-Admit (%)	377 (10.6)	282 (9.86%)	26 (10.6%)	95 (9.6%)	69 (9.16%)	1.3 (-3.1, 5.7)	1.0 (-1.5, 3.4)	0.4 (-4.4, 5.1)
New diagnosis of dementia (%)	266 (6.9%)	108 (3.8%)	52 (21.2%)	158 (15.9%)	106 (14.1%)	17.5 (12.3, 22.7)	12.2 (9.8, 14.6)	5.3 (-0.3, 10.9)
New discharge to LTC (%)	160 (4.1)	74 (2.6%)	21 (8.6%)	86 (8.7%)	65 (8.6%)	6.0 (2.3, 9.6)	6.1 (4.2, 7.9)	0.1 (-3.8, 4.0)

Chart review = CHART-DEL; ICD-10-CA = ICD administrative data coding; IQR = inter-quartile range; SD = standard deviation; CI = confidence interval; OT = occupational therapy; PT = physiotherapy; ICU = intensive care unit; LOS = length of stay in days; CAD = Canadian dollar currency; AMA = against medical advice; GIM = general internal medicine; LTC = long term care facility.

Compared to delirium cases identified by chart review, delirium cases identified by ICD-10 codes had greater median hospital length of stay by 2.7 days (95% CI: 0.4, 4.3) whereas they had lower mortality by 6.1% (95%CI: -1.8, -10.5) and lower ICU use by 5.3% (95%CI: -0.9, -9.8). It was uncertain whether median costs of care were greater for delirium cases identified by ICD-10 codes than chart review, with the estimate of difference being $1,844 but confidence intervals including no difference (95%CI: -602, 4019). Using chart review as the standard, ICD-10 codes had specificity of 99.8% (95%CI: 99.5–99.9%), sensitivity of 24.1% (95%CI: 21.5–26.8%), positive predictive value of 97.6% (95%CI: 94.6–98.9%) and negative predictive value of 79.2% (95%CI: 78.6–79.7%). As shown in Table 4, age over 80, male gender, and Charlson Comorbidity Index greater than 2 were associated with misclassification of delirium (i.e. delirium present by chart review, but absent by administrative data (n = 753) or present by administrative data, but absent by chart review (n = 6)).

**Table 4:** Factors associated with incorrect ICD-10 code identification of delirium.

Factor	Prevalence N (%)	Incorrect ICD-10 Identification N (%)		Adjusted Odds Ratio (95% CI)	Unadjusted Odds Ratio (95% CI)
		Factor Present	Factor Absent		
Age > = 80 years	1356 (35.1)	376/1356 (27.7)	383/2503 (15.3)	1.92 (1.62, 2.28)	2.12 (1.81–2.50)
Gender (M)	1913 (49.6)	409/1913 (21.4)	350/1946 (18.0)	1.30 (1.10, 1.54)	1.24 (1.06, 1.45)
Charlson comorbidity index (>2)	1633 (42.3)	422/1633 (25.8)	337/2226 (15.1)	1.58 (1.31, 1.90)	1.95 (1.66, 2.29)
Pre-Existing Dementia	67 (1.7)	19/67 (28.4)	740/3792 (19.5)	0.96 (0.54, 1.64)	1.63 (0.93, 2.75)

The models include all baseline characteristics (age, gender, LAPS, Charlson comorbidity index, pre-existing dementia) with incorrect ICD-10 identification (Y/N) as the outcome. Incorrect ICD-10 identification refers to both false positives and false negatives; CI = confidence interval. Factors Present refers to the proportion of patients who had delirium status misclassified by ICD-10 code among those with the factor of interest present (e.g., among the 67 patients with pre-existing dementia, 28.4% had delirium misclassified). Factors Absent refers to the proportion of patients who had delirium status misclassified by ICD-10 code among those without the factor of interest present (e.g., among the 2,792 patients without pre-existing dementia, 19.5% had delirium misclassified).

## Discussion

To assess delirium rates and populations for quality improvement and health services research, we need a reliable and scalable method for case identification. In this study, we compared routinely collected administrative data with a validated but resource-intensive chart review method for identifying delirium. We found that, although ICD-10 codes were highly specific, they substantially underreported the prevalence of delirium in hospitalized medical patients and missed approximately three-quarters of those identified by the chart review method. This suggests that studies and quality improvement (QI) projects which rely on administrative data alone for delirium identification likely miss most patients with delirium. Those that use administrative data to estimate costs and resource use associated with delirium would substantially underestimate the true burdens of delirium, and those that report outcomes would underestimate mortality and ICU admission, which is particularly important as delirium is known to be associated with death and critical illness [12,34]. Moreover, while there have been attempts to develop data-driven and machine learning approaches to predict delirium in hospital, these methods often use administrative data to train and test their models, which then replicates these limitations of identification through administrative data [35–38]

Importantly, our study demonstrates that there are differences between those identified with delirium by administrative coding and those identified by chart review. Administrative data was more likely to miss delirium in men, patients older than 80 years, and those with greater comorbidity. While we are not able to determine the mediating factors leading to these differences, previous research has demonstrated that documentation of features of the hyperactive subtype of delirium impacts administrative coding [18]. This discordance may also reflect differences in documentation of symptoms and behaviour across gender and illness severity groups. The observation that delirium is more likely to be misclassified by administrative data in patients over 80 years of age may indicate that coders struggle to distinguish between the acute mental status changes in delirium and chronic age-related cognitive changes, which is unsurprising given that this distinction can be challenging for clinicians.

Individuals with delirium identified by administrative data or chart review were more likely to have allied health assessments (OT or PT), specialist consultations (psychiatry and geriatric medicine), and cognitive testing. However, it appears that a greater proportion of those identified by administrative data had allied health and specialist consultations compared to those identified by chart review. From this study, we are not able to determine the directionality of this relationship. For example, these assessments may reflect recognition of a change in mental status or they may assist in identifying delirium when present, improving identification by both chart review and administrative data coding.

### Strengths

This study is novel in terms of its size, use of CHART-DEL which is a validated method of chart review, and its focus on medical inpatients which represent the largest proportion of admissions and hospital bed days at the hospitals studied. While delirium research often focuses on surgical and intensive care populations [18,39], medical ward patients are at high risk of delirium and its negative impacts. Our study includes multiple sites, including both academic and community hospitals with similar delirium rates across sites.

### Limitations

Although we did not include comparison to clinical assessment or screening, the proportion of patients identified by chart review as having delirium are similar to studies that have used these other methods [20,40]. Numerous tools for delirium screening have been validated, however, conducting and documenting these assessments is labor-intensive. It also requires significant institutional investment to support education, training, and audit and feedback [41–43]. Although routine screening using the Confusion Assessment Method (CAM) has since been implemented at several of the study sites, this was not conducted regularly during the study period [44]. Of note, despite twice daily screens by nursing staff and extensive staff training and education, delirium rates determined using the CAM at one study site were found to be lower than those determined by both chart review and administrative codes (3% unpublished data, personal communication). This is similar to other studies that have found that routine clinical screening under-reports up to three-quarters of cases compared to clinical assessments for research [41,45–47].

Previous studies comparing chart review to high-quality clinical assessments for research demonstrate that hypoactive delirium is more likely to be missed by chart review [48]. Inouye et al. (2005) concluded that the CHART-DEL method is appropriate to measure the impact of quality improvement initiatives and institutional “report cards” for delirium [23]. However, this method relies on documentation of delirium signs and symptoms in the chart and, when these continue to be under-reported or poorly documented, then delirium may not be identified. The chart review method may also result in false positive identification of delirium in patients with a higher baseline risk of delirium (e.g. those with dementia, severe illness). Performing regular chart reviews or reviewing large numbers of charts to track delirium rates also proves challenging from a human resources perspective, as each one can take a trained individual a substantial amount of time (15–60 minutes per chart) to complete. Nevertheless, the chart review method has been well-validated, is more feasible to implement, and does have some strengths above cross-sectional clinical assessments alone, such as the identification of symptoms that occurred at night. The most accurate method for determination of delirium combines clinical assessment with other sources of information, including collateral from informants and chart review.

## Conclusions

Our study adds to the delirium literature by characterizing populations identified through administrative diagnosis codes and a validated chart review method. Understanding who is captured by each identification method is important for several reasons. At both an institutional and health system level, determining rates through administrative data is faster and less costly. However, the risk of under-diagnosis and misclassification of delirium with administrative data is high. Relying on administrative data alone for quality improvement initiatives may risk over- or under-estimating the effectiveness of delirium interventions. Moreover, at a system level, using administrative data alone will also significantly underestimate the burden of delirium in terms of total days in hospital, mortality, ICU admission, and attributable costs.

Improvements in low-cost approaches to identify delirium are urgently needed. Novel approaches, including machine learning, using more aspects of administrative, electronic clinical data, and medical records have the potential to improve accuracy of identification while being faster and less costly than chart review.

## Supporting information

S1 Appendix ICD-10 codes used. (DOCX)

S2 Appendix Baseline and outcome characteristics association with delirium as determined by chart review. (DOCX)

S1 File PLOS ONE clinical studies checklist. (DOCX)

S2 File STROBE statement—checklist of items that should be included in reports of observational studies. (DOCX)
