# Supplementary material for: Characterizing medical patients with delirium: A cohort study comparing ICD-10 codes and a validated chart review method
Source: PLoS One. 2024 May 13;19(5):e0302888. doi: 10.1371/journal.pone.0302888 (PMC11090329; doi:10.1371/journal.pone.0302888)
Supplement: S1 Appendix — (DOCX) [file pone.0302888.s001.docx]

Appendix 1: ICD-10 Codes Used

| **Narrow Definition** | **Broad Definition**  **(below plus narrow definition codes)** |
| --- | --- |
| F05 Delirium | A812 Progressive multifocal leukoencephalopathy |
| F10121 Alcohol abuse with intoxication delirium | E512 Wernicke's encephalopathy |
| F10221 Alcohol dependence with intoxication delirium | G0430 Acute necrotizing hemorrhagic encephalopathy, unspecified |
| F10231 Alcohol dependence with withdrawal delirium | G0431 Post-infectious acute necrotizing hemorrhagic encephalopathy |
| F10921 Alcohol use, unspecified with intoxication delirium | G0432 Post-immunization acute necrotizing hemorrhagic encephalopathy |
| F11121 Opioid abuse with intoxication delirium | G0439 Other acute necrotizing hemorrhagic encephalopathy |
| F11221 Opioid dependence with intoxication delirium | G92 Toxic encephalopathy |
| F11921 Opioid use, unspecified with intoxication delirium | G9340 Encephalopathy, unspecified |
| F12121 Cannabis abuse with intoxication delirium | G9341 Metabolic encephalopathy |
| F12221 Cannabis dependence with intoxication delirium | G9349 Other encephalopathy |
| F12921 Cannabis use, unspecified with intoxication delirium | I673 Progressive vascular leukoencephalopathy |
| F13121 Sedative, hypnotic or anxiolytic abuse with intoxication delirium | I674 Hypertensive encephalopathy |
| F13221 Sedative, hypnotic or anxiolytic dependence with intoxication delirium | I6783 Posterior reversible encephalopathy syndrome |
| F13231 Sedative, hypnotic or anxiolytic dependence with withdrawal delirium | J1081 Influenza due to other identified influenza virus with encephalopathy |
| F13921 Sedative, hypnotic or anxiolytic use, unspecified with intoxication delirium | J1181 Influenza due to unidentified influenza virus with encephalopathy |
| F13931 Sedative, hypnotic or anxiolytic use, unspecified with withdrawal delirium | P9160 Hypoxic ischemic encephalopathy, unspecified |
| F14121 Cocaine abuse with intoxication with delirium | P9161 Mild hypoxic ischemic encephalopathy |
| F14221 Cocaine dependence with intoxication delirium | P9162 Moderate hypoxic ischemic encephalopathy |
| F14921 Cocaine use, unspecified with intoxication delirium | P9163 Severe hypoxic ischemic encephalopathy |
| F15121 Other stimulant abuse with intoxication delirium |  |
| F15221 Other stimulant dependence with intoxication delirium |  |
| F15921 Other stimulant use, unspecified with intoxication delirium |  |
| F16121 Hallucinogen abuse with intoxication with delirium |  |
| F16221 Hallucinogen dependence with intoxication with delirium |  |
| F16921 Hallucinogen use, unspecified with intoxication with delirium |  |
| F18121 Inhalant abuse with intoxication delirium |  |
| F18221 Inhalant dependence with intoxication delirium |  |
| F18921 Inhalant use, unspecified with intoxication with delirium |  |
| F19121 Other psychoactive substance abuse with intoxication delirium |  |
| F19221 Other psychoactive substance dependence with intoxication delirium |  |
| F19231 Other psychoactive substance dependence with withdrawal delirium |  |
| F19921 Other psychoactive substance use, unspecified with intoxication with delirium |  |
| F19931 Other psychoactive substance use, unspecified with withdrawal delirium |  |

ICD, International Classification of Diseases.
