# Supplementary material for: Characterizing medical patients with delirium: A cohort study comparing ICD-10 codes and a validated chart review method
Source: PLoS One. 2024 May 13;19(5):e0302888. doi: 10.1371/journal.pone.0302888 (PMC11090329; doi:10.1371/journal.pone.0302888)
Supplement: S2 Appendix — (DOCX) [file pone.0302888.s002.docx]

Appendix 2: Baseline and Outcome Characteristics Association with Delirium as Determined by Chart Review

|  | Overall Sample | Delirium  + chart review | Delirium  - chart review | p | Standard Mean Difference |
| --- | --- | --- | --- | --- | --- |
| N | 3859 | 992 | 2867 |  |  |
| Age  Median (IQR) | 73.00  (57.00, 84.00) | 80.00  (69.00, 87.00) | 69.00  (53.00, 81.00) | <0.001 | 0.616 |
| Gender Male (%) | 1913 (49.6) | 507 (51.1) | 1406 (49.0) | 0.277 | 0.041 |
| LAP Score Mean (SD) | 20.48 (17.56) | 25.41  (19.42) | 18.77  (16.54) | <0.001 | 0.369 |
| Charlson comorbidity index (%)  0  1  2+ | 1602 (41.5)  624 (16.2)  1633 (42.3) | 309 (31.1)  139 (14.0)  544 (54.8) | 1293 (45.1)  485 (16.9)  1089 (38.0) | <0.001 | 0.348 |
| Pre-Admission Dementia (%) | 67  (1.7) | 33  (3.3) | 34  (1.2) | <0.001 | 0.145 |
| LOS (days)  Median (IQR) | 4.8  (2.5, 9.6) | 8.8  (4.5, 17.4) | 4.1  (2.0, 7.6) | <0.001 | 0.539 |
| OT Assessment | 1261 (32.7%) | 515  (51.9%) | 746  (26.1%) | <0.001 | 0.549 |
| PT Assessment | 1532 (39.8%) | 591  (59.6%) | 941  (32.9%) | <0.001 | 0.556 |
| Geriatric Medicine Consult | 203 (5.3%) | 128 (12.9%) | 75 (2.6%) | <0.001 | 0.392 |
| Geriatric Psychiatry Consult | 76 (2.0%) | 37 (3.7%) | 39 (1.4%) | <0.001 | 0.151 |
| Psychiatry Consult | 187 (4.9%) | 78 (7.9%) | 109 (3.8%) | <0.001 | 0.174 |
| Cognitive Testing Performed | 360 (9.3%) | 195 (19.7%) | 165 (5.8%) | <0.001 | 0.426 |
| ICU Admission (%) | 314 (8.1%) | 154 (15.5%) | 160 (5.6%) | <0.001 | 0.327 |
| ICU LOS (days)  Median (IQR) | 4.0  (1.7, 8.2) | 5.3  (2.0, 10.8) | 2.7  (1.5, 6.2) | <0.001 | 0.539 |
| Total cost (CAD)  Median (IQR) | 5049.3 (2650.8, 9968.6) | 9180.0 (4552.6, 19411.0) | 4199.3 (2290.5, 7885.8) | <0.001 | 0.489 |
| Mortality (%) | 258 (6.7%) | 154 (15.5%) | 104 (3.6%) | <0.001 | 0.412 |
| AMA Discharge (%) | 57 (1.5%) | 6 (0.6%) | 51 (1.8%) | 0.013 | 0.109 |
| 30 Day GIM  Re-Admit (%) | 377 (10.6%) | 95 (11.3%) | 282 (10.3%) | 0.473 | 0.031 |
| New diagnosis of dementia (%) | 264 (6.9) | 158 (15.9%) | 106 (3.7%) | <0.001 | 0.420 |
| New discharge to LTC (%) | 160 (4.2%) | 86 (8.7%) | 74 (2.6%) | <0.001 | 0.266 |

IQR - inter-quartile range; LAP – laboratory-based acute physiology; LOS – length of stay; OT – occupational therapy; PT – physiotherapy; ICU – intensive care unit; AMA – against medical advice; GIM – general internal medicine; LTC – long term care
