# Supplementary material for: Characterizing medical patients with delirium: A cohort study comparing ICD-10 codes and a validated chart review method
Source: PLoS One. 2024 May 13;19(5):e0302888. doi: 10.1371/journal.pone.0302888 (PMC11090329; doi:10.1371/journal.pone.0302888)
Supplement: S2 File — (DOCX) [file pone.0302888.s004.docx]

STROBE Statement—checklist of items that should be included in reports of observational studies

|  | Item No. | Recommendation | Page  No. | Relevant text from manuscript |
| --- | --- | --- | --- | --- |
| **Title and abstract** | 1 | (*a*) Indicate the study’s design with a commonly used term in the title or the abstract | 1 | A cohort study comparing ICD-10 codes and a validated chart review method |
|  |  | (*b*) Provide in the abstract an informative and balanced summary of what was done and what was found | 2 | Delirium was identified in 6.3% of admissions by ICD-10-CA codes compared to 25.7% by chart review. Using chart review as the reference standard, ICD-10-CA codes for delirium had sensitivity 24.1% (95%CI: 21.5-26.8%), specificity 99.8% (95%CI: 99.5-99.9%), positive predictive value 97.6% (95%CI: 94.6-98.9%), and negative predictive value 79.2% (95%CI: 78.6-79.7%). |
| Introduction | | | |  |
| Background/rationale | 2 | Explain the scientific background and rationale for the investigation being reported | 3 | Delirium is a common, preventable, and treatable, neurocognitive disorder, characterized by acute onset of fluctuating mental status, psychomotor disturbance and hallucinations ^1^. It affects up to 50% of hospitalized adults over the age of 65 and is associated with numerous negative outcomes in hospital including increased mortality, longer stays, and higher health care costs ^2,3^. Given its lower cost and ready accessibility, administrative data has been recommended for evaluating delirium rates according to quality standards in Australia and Canada ^9,10^. This method is also used in population-based and health services research ^12,13^ . However, it is recognized that administrative data substantially underestimate the frequency of delirium compared to case ascertainment by either chart review or clinical assessment ^12,14–22^. While some studies have compared administrative coding to clinical assessment, there are limited data comparing administrative codes to a validated chart review method ^17–19,23^. |
| Objectives | 3 | State specific objectives, including any prespecified hypotheses | 3-4 | From a system perspective, understanding who is, or is not, captured by each method may be of more use than understanding the accuracy of each detection method. With this knowledge, those working in healthcare institutions can decide which method of delirium identification is most appropriate for their quality assurance and improvement aims. In this study, we aimed to characterize and compare the populations of medical inpatients with delirium, as identified by administrative data and a chart review method. |
| Methods | | | |  |
| Study design | 4 | Present key elements of study design early in the paper | 4 | We conducted a retrospective chart review study, randomly selecting 3881 hospital admissions from four healthcare institutions (six hospitals) in Toronto, Ontario. |
| Setting | 5 | Describe the setting, locations, and relevant dates, including periods of recruitment, exposure, follow-up, and data collection | 4 | Participating institutions were St. Michael’s Hospital (SMH), University Health Network (UHN: Toronto General Hospital and Toronto Western Hospital), Sunnybrook Health Sciences Centre (SBK), and Trillium Health Partners (THP: Credit Valley Hospital and Mississauga Hospital). Each hospital is part of the GEMINI research network which collects administrative and clinical data on all adult (>18 year of age) patients who are admitted to or discharged from general medicine services ^24,25^. These hospitals range in size from 433 to 1325 acute inpatient beds, with general internal medicine (GIM) patients accounting for 24% of hospital bed-days and 39% of admissions through the emergency department during the study period of April 1 2010 – March 20 2015 ^24^. |
| Participants | 6 | (*a*) *Cohort study*—Give the eligibility criteria, and the sources and methods of selection of participants. Describe methods of follow-up  *Case-control study*—Give the eligibility criteria, and the sources and methods of case ascertainment and control selection. Give the rationale for the choice of cases and controls  *Cross-sectional study*—Give the eligibility criteria, and the sources and methods of selection of participants | 4,7 | These hospitals range in size from 433 to 1325 acute inpatient beds, with general internal medicine (GIM) patients accounting for 24% of hospital bed-days and 39% of admissions through the emergency department during the study period of April 1 2010 – March 20 2015 ^24^.  Of the 3881 randomly selected patient records, the final sample included 3859 patient admissions across six hospitals. |
|  |  | (*b*) *Cohort study*—For matched studies, give matching criteria and number of exposed and unexposed  *Case-control study*—For matched studies, give matching criteria and the number of controls per case |  |  |
| Variables | 7 | Clearly define all outcomes, exposures, predictors, potential confounders, and effect modifiers. Give diagnostic criteria, if applicable | 6 | Administrative health data were linked with clinical data extracted from hospital information systems ^24^. Demographic variables included age and gender. Clinical variables included the laboratory-based acute physiology score (LAPS), Charlson Comorbidity Index (CCI), and a pre-admission diagnosis of dementia ^29–31^. Processes of care variables included length of stay, admission to an intensive care unit (ICU), and ICU length of stay (LOS). Outcome variables included total cost of hospitalization (direct and indirect costs from the emergency department and inpatient portions of the hospital visit), mortality, 30-day readmission to general internal medicine (GIM) at any participating hospital, new diagnosis of dementia at discharge, and a new discharge destination of a long term care (LTC) facility ^32^  In addition to variables extracted from these electronic systems, processes of care variables were also coded during the process of manual chart review. These included specialist physician consultation (geriatric medicine, psychiatry and geriatric psychiatry), allied health (physiotherapy and occupational therapy) assessment, and completion of neurocognitive screening. |
| Data sources/ measurement | 8* | For each variable of interest, give sources of data and details of methods of assessment (measurement). Describe comparability of assessment methods if there is more than one group | *4,5* | GEMINI collects and links administrative and clinical data for patients admitted to or discharged from general medicine services ^24,26^. The administrative data include data reported by participating hospitals to the Canadian Institute for Health Information (CIHI) Discharge Abstract Database (DAD): demographic characteristics, diagnoses, interventions, discharge destinations, and resource use. For each hospitalization up to 25 diagnoses are recorded at the time of discharge in the DAD.  The CHART-DEL method, which has a sensitivity of 74% and specificity of 83% compared to clinical assessment, was used to identify delirium during hospitalization ^23^. Information about and documentation of acute changes in mental status were transcribed verbatim, as well as improvements in mental state. |
| Bias | 9 | Describe any efforts to address potential sources of bias | n/a |  |
| Study size | 10 | Explain how the study size was arrived at | 7 | The sample size was selected based on the feasibility of chart review, with an estimated 30-60 minutes required per admission. |

Continued on next page

| Quantitative variables | 11 | Explain how quantitative variables were handled in the analyses. If applicable, describe which groupings were chosen and why | n/a |  |
| --- | --- | --- | --- | --- |
| Statistical methods | 12 | (*a*) Describe all statistical methods, including those used to control for confounding |  | 7 Descriptive statistics were used to compare demographic, clinical, service use, and outcome variables between those identified by chart review and those identified through administrative data. Given the sample size, standardized differences (SD) were used to assess statistical significance, with differences of greater than 0.1 considered significant ^33^. Baseline factors associated with incorrect identification of delirium by administrative data (false positives and false negatives) were examined using unadjusted and adjusted logistic regression models, including patient demographic and clinical variables. A random subsample of 5% of charts were coded by two abstractors to calculate inter-rater reliability using a kappa score. |
|  |  | (*b*) Describe any methods used to examine subgroups and interactions | n/a |  |
|  |  | (*c*) Explain how missing data were addressed | n/a |  |
|  |  | (*d*) *Cohort study*—If applicable, explain how loss to follow-up was addressed  *Case-control study*—If applicable, explain how matching of cases and controls was addressed  *Cross-sectional study*—If applicable, describe analytical methods taking account of sampling strategy | n/a |  |
|  |  | (*e*) Describe any sensitivity analyses |  |  |
| Results | | | | |
| Participants | 13* | (a) Report numbers of individuals at each stage of study—eg numbers potentially eligible, examined for eligibility, confirmed eligible, included in the study, completing follow-up, and analysed | 8 | Of the 3881 randomly selected patient records, the final sample included 3859 patient admissions across six hospitals. Twenty-two were excluded because we were unable to locate patient records (Figure 1). |
|  |  | (b) Give reasons for non-participation at each stage | 8 | Figure 1 |
|  |  | (c) Consider use of a flow diagram |  |  |
| Descriptive data | 14* | (a) Give characteristics of study participants (eg demographic, clinical, social) and information on exposures and potential confounders | 8 | As shown in Table 1, the sample was 49.6% male and the median age was 73 years (IQR: 57-84). The mean LAPS was 20.48 (SD: 17.56). Similar proportions of the sample had either low (41.5%, score of 0) or high (42.3%, score >2) levels of comorbidity as measured by the Charlson Comorbidity Index. Approximately 2% (N=67) had dementia noted as a pre-admission comorbidity ^31^. Delirium was identified in 992 (25.7%) patients by chart review, compared to 245 (6.3%) using ICD-10 codes. In only six cases (0.16%) was delirium identified by ICD-10 codes, but not by chart review (Appendix 2 shows sample characteristics by chart review delirium identification). |
|  |  | (b) Indicate number of participants with missing data for each variable of interest | n/a |  |
|  |  | (c) *Cohort study*—Summarise follow-up time (eg, average and total amount) | 10 | Hospital length-of-stay and costs of care were greater for patients identified as having delirium by ICD-10 codes (median 11.45 days, IQR 5.83-22.17; median $11,023.60, IQR 5,125.00 – 22,223.10) or chart review (median 8.8 days, IQR 4.5-17.4; median $9,180.00, IQR 4,552.60-19,411.00) compared to patients without delirium (median 4.08 days, IQR 2.04-7.62; median $4,199.30, IQR 2,290.45-7,885.79). Similarly, inpatient mortality and ICU admission were greater in patients with delirium identified by ICD-10 codes (mortality 9.4%, ICU 10.2%) or chart review (mortality 15.5%, ICU 15.5%) compared to patients without delirium (mortality 3.6%, ICU 5.6%). A new diagnosis of dementia and new placement in long-term care were also greater in patients with delirium identified by either method compared to patients without delirium (Table 3). |
| Outcome data | 15* | *Cohort study*—Report numbers of outcome events or summary measures over time | *8* | Delirium was identified in 992 (25.7%) patients by chart review, compared to 245 (6.3%) using ICD-10 codes. In only six cases (0.16%) was delirium identified by ICD-10 codes, but not by chart review (Appendix 2 shows sample characteristics by chart review delirium identification). The use of the chart review method detected 753 more patients than those identified by ICD-10 codes alone. |
|  |  | *Case-control study—*Report numbers in each exposure category, or summary measures of exposure |  |  |
|  |  | *Cross-sectional study—*Report numbers of outcome events or summary measures |  |  |
| Main results | 16 | (*a*) Give unadjusted estimates and, if applicable, confounder-adjusted estimates and their precision (eg, 95% confidence interval). Make clear which confounders were adjusted for and why they were included | 13 | Using chart review as the standard, ICD-10 codes had specificity of 99.8% (95%CI: 99.5-99.9%), sensitivity of 24.1% (95%CI: 21.5-26.8%), positive predictive value of 97.6% (95%CI: 94.6-98.9%) and negative predictive value of 79.2% (95%CI: 78.6-79.7%). As shown in Table 4, age over 80, male gender, and Charlson Comorbidity Index greater than 2 were associated with misclassification of delirium (i.e. delirium present by chart review, but absent by administrative data (n=753) or present by administrative data, but absent by chart review (n=6)). |
|  |  | (*b*) Report category boundaries when continuous variables were categorized |  |  |
|  |  | (*c*) If relevant, consider translating estimates of relative risk into absolute risk for a meaningful time period |  |  |

Continued on next page

| Other analyses | 17 | Report other analyses done—eg analyses of subgroups and interactions, and sensitivity analyses |  |  |
| --- | --- | --- | --- | --- |
| Discussion | | | | |
| Key results | 18 | Summarise key results with reference to study objectives | 15 | To assess delirium rates and populations for quality improvement and health services research, we need a reliable and scalable method for case identification. In this study, we compared routinely collected administrative data with a validated but resource-intensive chart review method for identifying delirium. We found that although ICD-10 codes were highly specific, they substantially underreport the prevalence of delirium in hospitalized medical patients and missed approximately three-quarters of those identified by the chart review method. This suggests that studies and quality improvement (QI) projects that rely on administrative data alone for delirium identification likely miss most patients with delirium. Moreover, those that use administrative data to estimate costs and resource use associated with delirium would substantially underestimate the true burdens of delirium, and those that report outcomes would underestimate mortality and ICU admission, which is particularly important as delirium is known to be associated with death and critical illness.^12,34^  Importantly, our study demonstrates that there are differences between those identified with delirium by administrative coding and those identified by chart review. Administrative data was more likely to miss delirium in men, patients older than 80 years, and those with greater comorbidity. |
| Limitations | 19 | Discuss limitations of the study, taking into account sources of potential bias or imprecision. Discuss both direction and magnitude of any potential bias | 16-17 | Although we did not include comparison to clinical assessment or screening, the proportion of patients identified by chart review as having delirium are similar to studies that have used these other methods ^20,36^. Numerous tools for delirium screening have been validated, however, conducting and documenting these assessments is labor-intensive. It also requires significant institutional investment to support education, training, and audit and feedback ^37–39^. Although routine screening using the Confusion Assessment Method (CAM) has since been implemented at several of the study sites, this was not conducted regularly during the study period ^40^. Of note, despite twice daily screens by nursing staff and extensive staff training and education, delirium rates determined using the CAM at one study site were found to be lower than those determined by both chart review and administrative codes (3% unpublished data, personal communication). This is similar to other studies that have found that routine clinical screening under-reports up to three-quarters of cases compared to clinical assessments for research ^37,41–43^. |
| Interpretation | 20 | Give a cautious overall interpretation of results considering objectives, limitations, multiplicity of analyses, results from similar studies, and other relevant evidence | 17-18 | Our study adds to the delirium literature by characterizing populations identified through administrative diagnosis codes and a validated chart review method. Understanding who is captured by each identification method is important for several reasons. At both an institutional and health-systems level, determining rates through administrative data is faster and less costly. However, the risk of under-diagnosis and misclassification of delirium with administrative data is high. Relying on administrative data alone for quality improvement initiatives may risk over- or under-estimating the effectiveness of delirium interventions. Moreover, at a system level, using administrative data alone will also significantly underestimate the burden of delirium in terms of total days in hospital, mortality, ICU admission, and attributable costs. |
| Generalisability | 21 | Discuss the generalisability (external validity) of the study results | 16 | This study is novel in terms of its size, use of CHART-DEL which is a validated method of chart review, and its focus on medical inpatients which represent the largest proportion of admissions and hospital bed days at the hospitals studied. While delirium research often focuses on surgical and intensive care populations ^18,35^, medical ward patients are at high risk of delirium and its negative impacts. Our study includes multiple sites, including both academic and community hospitals with similar delirium rates across sites.  Although we did not include comparison to clinical assessment or screening, the proportion of patients identified by chart review as having delirium are similar to studies that have used these other methods |
| Other information | |  | | |
| Funding | 22 | Give the source of funding and the role of the funders for the present study and, if applicable, for the original study on which the present article is based |  | Funding information was entered into the Editorial Manager form. |

*Give information separately for cases and controls in case-control studies and, if applicable, for exposed and unexposed groups in cohort and cross-sectional studies.

**Note:** An Explanation and Elaboration article discusses each checklist item and gives methodological background and published examples of transparent reporting. The STROBE checklist is best used in conjunction with this article (freely available on the Web sites of PLoS Medicine at http://www.plosmedicine.org/, Annals of Internal Medicine at http://www.annals.org/, and Epidemiology at http://www.epidem.com/). Information on the STROBE Initiative is available at www.strobe-statement.org.
